# More Than One Voice: Strengthening Organizational Readiness for Lived Experience Integration in Early Psychosis Services

**DOI:** 10.1111/hex.70944

**Published:** 2026-11-01

**Authors:** Stephania L. Hayes, Katherine C. Sanford, Alishia Dauterive, Naomi R. Jones, Shannon Pagdon, Maureen F. McSweeney, Josephine Lumaquin, Mark Savill, Tara Niendam

**Affiliations:** ^1^ Department of Psychiatry & Behavioral Sciences University of California, Davis Sacramento California USA; ^2^ Department of Psychiatry & Behavioral Sciences Stanford University School of Medicine Stanford California USA

**Keywords:** co‐production, early psychosis, implementation, lived experience, mental health services, organizational readiness, service user involvement

## Abstract

Knowledge derived from personal experience of mental health service use, by way of individuals with lived and living experience, is increasingly incorporated into mental health programs. Yet organizational readiness to support meaningful integration often lags behind intent. This paper offers a case analysis of the first 2 years of a Lived Experience Integration (LEI) Team embedded within a statewide early psychosis training and technical assistance network. Using Organizational Readiness for Change as a conceptual framework, this paper describes how leadership from people with lived experience contributed to training development, consultation, advisory structures, and workforce design, while navigating evolving infrastructure and the relational and interpretive demands of lived experience roles. Practical lessons are highlighted for building readiness to support durable lived experience integration in complex service systems.

Lived Experience or Public Contribution: This manuscript describes work that was initiated and led with substantial involvement from individuals with lived experience of psychosis‐spectrum conditions and mental health service use. Lived experience‐identified contributors built the LEI Team under discussion, participated in consultation and training activities, advised on organizational tensions described in this case analysis, and were fully responsible for the conceptualization and writing of this manuscript.

## 1 Introduction

Incorporating lived experience perspectives into mental health systems is increasingly recognized as essential for improving service quality and strengthening relevance [1], including how treatment and recovery are understood. A growing body of scholarship highlights the contributions that service users make as educators, consultants, researchers, and leaders, and documents the organizational and systemic barriers that limit their influence in practice [2, 3, 4, 5]. Programs frequently express commitment to involving people with lived experience, yet few have the infrastructure to implement these roles in a way that is sustainable and equitable.

This paper presents a case analysis of the first 2 years of the Lived Experience Integration (LEI) Team at EPI‐CAL, a statewide early psychosis intervention training and technical assistance (TTA) network. By examining how this team emerged and navigated structural constraints, we share what it takes to implement lived experience‐informed innovation inside a fast‐paced, grant‐driven academic environment. Organizational Readiness for Change (ORC) provides a useful conceptual lens for understanding how motivation, collective efficacy, resources, and contextual factors shape the conditions under which lived experience integration can take root. Our aim is to offer concrete lessons for programs seeking to build capacity responsibly, particularly those operating in academic medical settings where timelines are tight, roles evolve rapidly, and structures for meaningfully sharing power are still emerging.

## 2 Background: Lived Experience Integration and Organizational Conditions

Service user involvement in care planning is a widely‐stated goal of modern mental health systems, though research continues to document substantial gaps between policy expectations and routine practice [6, 7]. Efforts to embed lived experience in mental health systems take multiple forms [8], including peer support provider roles [9, 10, 11], advisory councils [12], co‐produced research [1, 13, 14, 15, 16, 17, 18], and training initiatives that foreground service user perspectives [19, 20]. Several well‐documented challenges parallel tensions in diversity, equity, inclusion, and accessibility initiatives in health and academic settings [21, 22]. Work drawing on lived experience involves substantial emotional and narrative labour that remains undervalued or invisible within institutional contexts [20], and roles are structurally fragile when employment conditions and supports are unclear [23]. In addition, representation poses a persistent challenge, as individuals who are recruited into institutional roles frequently reflect only a narrow range of service user perspectives [13, 19, 24]. When involvement is symbolic rather than structurally supported, tokenism can emerge, limiting both influence and impact [25, 26, 27]. These patterns suggest that integration is not achieved by placing people with lived experience ‘in the room’, but by creating organizational conditions where their knowledge determines what the room does. Therefore, successful integration is an organizational change process requiring shifts in culture, workflow, power‐sharing, and expectations. And in this sense, individuals with lived experience often assume leadership simply by entering systems that were not designed to include their knowledge.

To help organize these dynamics, we draw on Organizational Readiness for Change (ORC) [28], a widely‐used framework that conceptualizes readiness as an organizational state characterized by both (1) change commitment, defined as the collective resolve to pursue a specific change, and (2) change efficacy, the shared confidence in the organization's ability to implement that change (Figure 1). In ORC, readiness is affected by how organizational members value the change (change valence) and by their assessment of implementation demands. These appraisals are influenced by contextual factors such as organizational history, culture, incentives, and prior experience with change. Importantly, ORC treats readiness as change‐specific and potentially uneven across organizational units, with variability in commitment or efficacy indicating lower readiness.

**Figure 1:**
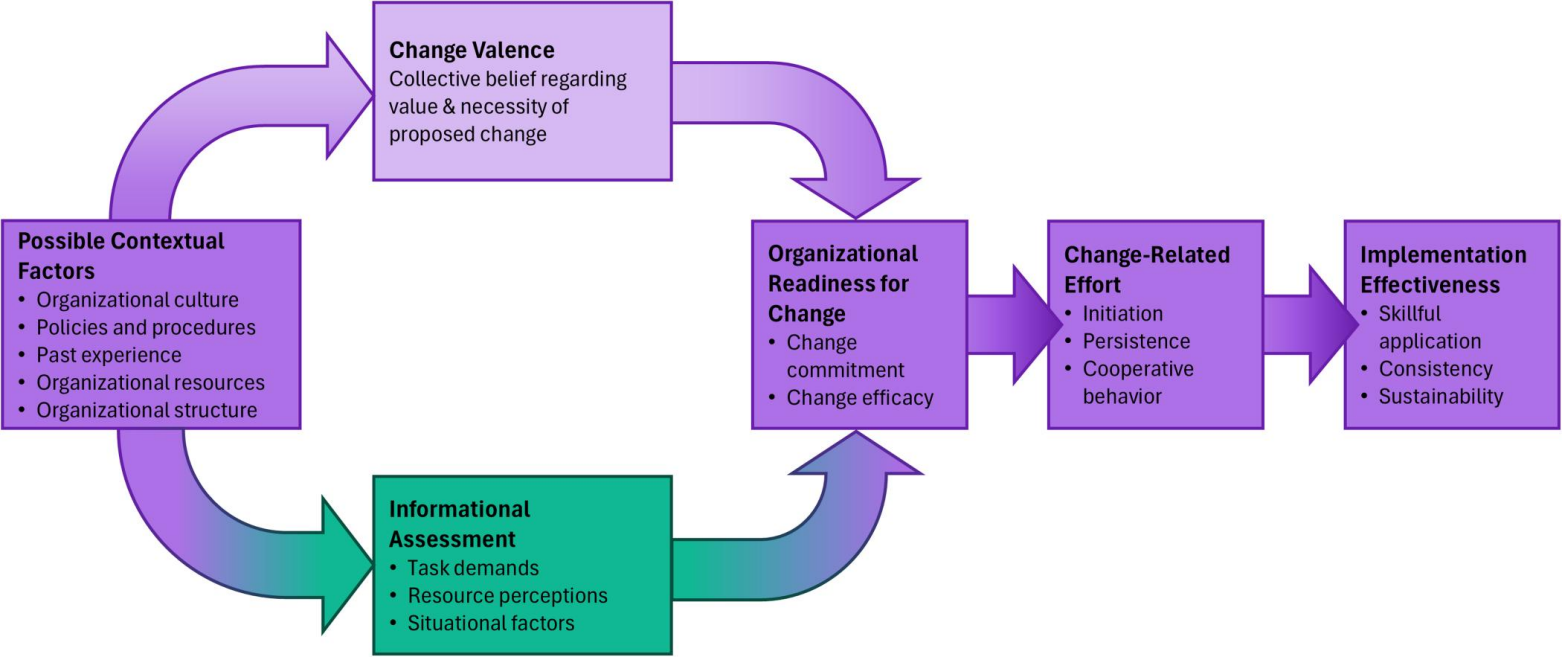
Organizational Readiness for Change framework, adapted from Weiner, 2009.

We apply Organizational Readiness for Change to understand why service user involvement initiatives thrive in some settings and struggle in others. Lived experience integration places new demands on organizations by requiring support and decision‐making structures that extend beyond customary clinical or academic routines. ORC helps illuminate where enthusiasm is high but infrastructure is underdeveloped, allowing us to examine what the organization *wanted to do*, what it was *prepared to do*, and what *had to be built*.

## 3 Program Context: EPI‐CAL and the TTA Ecosystem

EPI‐CAL is a statewide initiative housed at the University of California, Davis and, in collaboration with University of California, San Francisco and Stanford University, supports Coordinated Specialty Care (CSC) programs throughout California. EPI‐CAL began as a multi‐county learning healthcare network supported by county innovation funds, philanthropic partners such as One Mind, and state and federal funding. Initially focused on digital solutions to harmonize data collection across early psychosis programs, EPI‐CAL later evolved to provide training and technical assistance for CSC programs statewide.

The Training and Technical Assistance (TTA) program expanded through overlapping state and federal funding mechanisms and provides multiple supports to participating CSC clinics. These include liaison consultation, asynchronous role‐specific modules, live CSC trainings, statewide learning collaboratives, fidelity assessments, and training in a shared platform for measurement‐based care and outcomes monitoring. By mid‐2024, the network served 42 California counties (72%), with multiple CSC clinics operating in many counties.

During the first 2 years of implementation (2022–2024), the TTA program was organized into training teams aligned with core CSC components: assessment, medication management, psychotherapy, family services, outreach, supported education and employment, and group interventions. Learning Management and Fidelity teams supported statewide training delivery and monitoring (Figure 2). Personnel were distributed between three academic sites, and staffing and levels of effort changed as the program expanded, with some individuals contributing to multiple teams. At that time, efforts to incorporate people with relevant firsthand experience in EPI‐CAL's program leadership were still nascent, though the program had undertaken preliminary activities; for example, a mixed‐methods study invited CSC service users and family members to help set outcomes measurement priorities [29], and people with lived experience of mental health treatment (though not for psychosis) worked as junior researchers.

**Figure 2:**
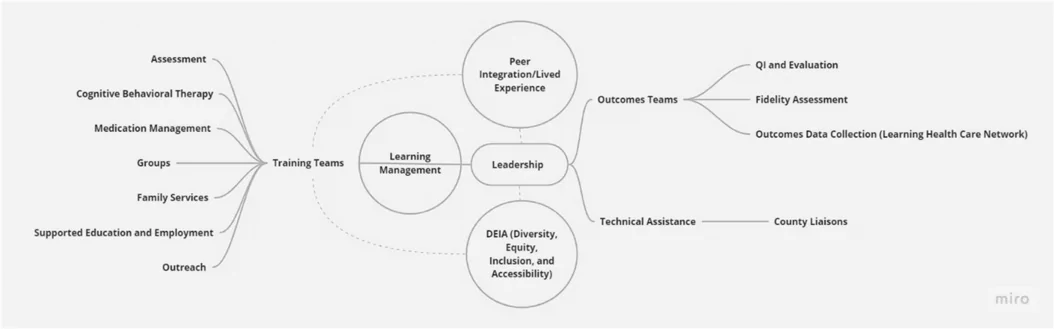
Organizational structure of EPI‐CAL during the initial phase of Lived Experience Integration Team implementation (2024).

## 4 LEI Team Origins: Change Commitment and Initiation

The LEI Team began when a postdoctoral scholar (first author) was asked to develop a mechanism for incorporating service user perspectives into EPI‐CAL's TTA activities. At an early strategic planning meeting, leadership identified one potential contribution as reviewing training content for language or framing that might be problematic from a service user perspective. Strong support for lived experience involvement reflected an organizational goal of bringing these perspectives into TTA program development and a shared belief that doing so was important and worthwhile (high change valence). At the same time, no formal process specified which TTA activities should involve lived experience expertise, when that involvement should occur, or what decision‐making authority it would carry. The initial plan centred on a single lived experience‐identified contributor working alongside clinicians and other TTA staff, but it became clear that meaningful integration could not be carried by a single individual. The need emerged for a group of people with lived experience who could collectively speak to the complexity of psychosis‐related trajectories and contribute expertise across many EPI‐CAL teams.

Acting on this recognition, the first author convened a small group of colleagues with lived experience of psychosis‐spectrum conditions (used here as an umbrella term, not a group identity) to establish a distinct unit inside the program. The initiative was positively received by TTA program leadership, and the three founding members together developed the initial team structure. Table 1 summarizes the core team's composition and the different funding and employment arrangements through which members participated. In contrast to common models in early psychosis systems that incorporate lived experience through peer support roles or advisory mechanisms, this group was intended to contribute across the program's core activities (Figure 2), with a mission to integrate lived experience into training development, research conceptualization, advisory activities, and consultation practices. Early efforts were guided by shared values of inclusivity, accountability, and a mindset for growth and learning, providing coherence at a time when formal role expectations had not yet been established. No formal evaluation plan or measures of implementation and impact were established at initiation, though the team tracked consultation requests and completed activities for operational purposes. As EPI‐CAL's first effort to formalize lived experience involvement at this level, the initiative required core structures to be developed concurrently with the work itself (Figure 3).

**Table 1:** Composition, employment arrangements, and relevant expertise of the Lived Experience Integration core leadership team during early implementation (2022–2024).

Team role	Employment arrangement	Dedicated hours/week	Relevant experience	Pathway to team	Period active
Founding member, identified lead	Postdoctoral scholar ⇒ faculty appointment	15	Clinical practice; research; peer support; peer‐led organization management	Referred through lived experience researcher network; hired for research/clinical expertise; advocacy work contributed to faculty transition	03/2022–06/2024
Founding member	Partner‐site staff supported through subaward	10	BA forensic psychology; early‐career researcher in MSW/PhD program; peer support	Recruited from team lead's professional network, incidentally hired at partner site	01/2023–08/2024
Founding member	Full‐time program staff	40	BA psychology; policy, legal, and advocacy experience	Recruited from professional conference	02/2023–present
Team member	Contractor, then hired as hybrid peer support supervisor/LEI Team staff	10 ⇒ 20	BA psychology, MPH (in progress); peer support; policy and advocacy; ancestral knowledge	Professional conference and peer support networks	10/2023–present
Team member	Affiliated CSC clinic staff with partial effort reassigned to LEI Team	5	BA psychology; first Peer Case Manager at clinic to receive state peer specialist certification	Via clinic where team lead provided supervision of peer support workers	10/2023–03/2024
Team member	Contractor	10	BA psychology (in progress); youth/student advocacy; peer support	Professional conference and community/advocacy networks	11/2023–06/2024

*Note:* The table describes membership during early implementation, organized in order of arrival and periods active are approximate. Roster does not include additional contractors or advisory council members who contributed to specific activities.

**Figure 3:**
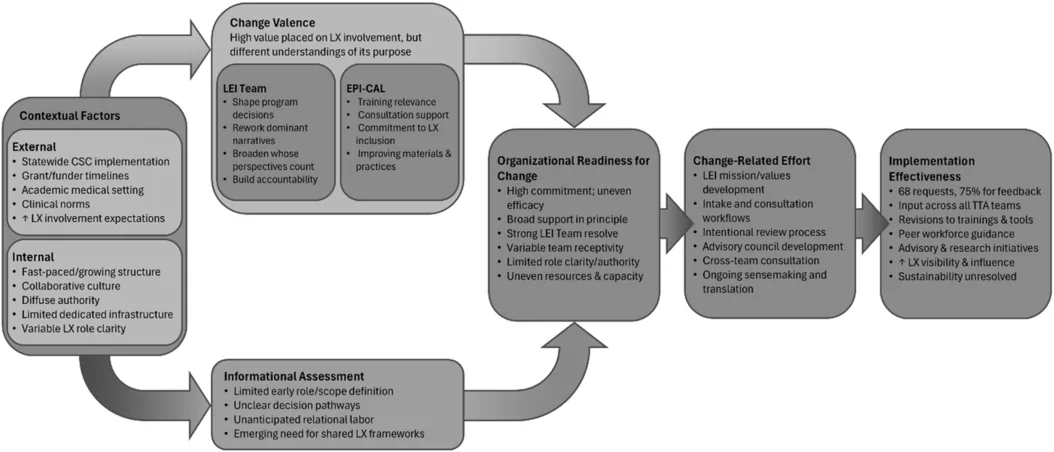
Organizational Readiness for Change constructs as manifested in Lived Experience Integration (LEI) Team implementation. LX, lived experience.

## 5 Contextual Factors: Organizational Structure and Resources

Organizational Readiness for Change highlights the importance of structural conditions (such as resources, workflows, and role clarity) in shaping readiness for an innovation. Although ORC emphasizes internal conditions, this work also unfolded as the TTA itself developed its statewide role. Within the TTA program, new county and state‐level requests emerged alongside ongoing consultation and fidelity work, creating pressure to prioritize immediate deliverables over longer‐term capacity building. Existing trainings were revised as new initiatives were added on short timelines, while the TTA responded to emerging issues and policy changes affecting CSC practice. This ongoing organizational learning created new settings in which lived experience perspectives were relevant. At the same time, diffuse reporting structures and overlapping supervisory lines across the three participating academic centres made it difficult to define where the Lived Experience Integration Team's expertise was best embedded.

**Foundations for Early Growth.** Early efforts unfolded during a period when the TTA program had access to several funding mechanisms and some flexibility for experimenting with new forms of leadership and innovative team design. A temporary COVID‐era grant allowed funding for the team to prepare job descriptions and preliminary infrastructure for strategic growth. Institutional technology such as teleconferencing and cloud‐based systems enabled collaboration across dispersed locations without a centralized physical space. Key staff also acted as informal champions, advocating for structural support and helping the team navigate research and external consultation opportunities.

**Missing Infrastructure.** Certain resource domains were limited or absent. The Lived Experience Integration Team did not have a dedicated budget line, stable hiring pathways, or job classifications that aligned with the distinct demands of roles in which lived experience was itself a form of expertise—an example of resource readiness trailing conceptual readiness under the Organizational Readiness for Change framework. Human resources were constrained, as existing staff took on responsibilities alongside their primary roles, typically without corresponding protected time. This included extra cross‐team consultation and development of new functions such as peer support training pathways. Administrative processes for bringing new members onto the team moved slowly, and once they joined, there was little structured orientation to the TTA program or the academic, grant‐funded environment. New contributors largely acquired this context by reading program materials and entering ongoing meetings and projects. This reliance on informal learning was particularly relevant as the team sought to recruit people with lived experience who might have little prior exposure to academic or grant‐funded settings.

There was not always a shared organizational understanding of the principles guiding lived experience involvement or a common interpretation of concepts central to service user‐centred care. For example, views differed on whether recovery from psychosis was possible and what recovery should mean in practice, including whether recovery‐oriented perspectives were appropriate for the Lived Experience Integration Team to educate about or advocate for. These differences affected how lived experience‐informed recommendations were received and made it harder to establish consistent conditions for the work. In the absence of organizational norms, the LEI Team frequently relied on individual relationships and case‐by‐case advocacy to move the work forward.

Formal support specific to lived experience‐oriented work was limited. The team lead provided weekly individual mentorship to several members, and team meetings offered space to work through difficult interactions. Members also sought informal consultation from experienced advocates outside the program. Within the greater organization, however, supervisory expertise was generally oriented toward academic or clinical roles. Lived experience integration raised different questions about peer practice and the professional use of personal experience, areas in which established mentorship capacity was limited.

**Implementation Shaped by Constraint.** Fractional roles and contracted work were common throughout the TTA program, although the LEI Team relied particularly heavily on contractors during its early growth. The temporary period of flexible funding described above created a narrow opportunity to expand the team, and contracting was the fastest available way to bring additional lived expertise into the program. This approach made early participation possible, but changes in funding disrupted continuity and limited the ability to sustain emerging activities. Turnover and split roles reduced shared context over time and constrained work that required sustained coordination or long‐range planning.

These staffing conditions shaped how lived experience perspectives entered program decision‐making. Because team members’ time and authority spanned roles and programs, their contributions were more readily incorporated in settings where decision pathways were immediate and locally controlled, and less so where decisions involved cross‐team coordination or extended follow‐through. In this way, the resource environment influenced the pace of implementation and the forms of participation that were feasible during the early phase.

## 6 Meta‐Work: Nature and Demands of LEI Roles

The Lived Experience Integration Team represented a significant organizational innovation that called for capabilities not previously developed. Much of its work relied on relational labour and narrative reframing that were unfamiliar in an academic system where influence typically flows through professional degrees and clinical licensure. Relational labour involved building and maintaining the working relationships needed for lived experience perspectives to enter collaborative decision‐making, particularly when those perspectives challenged established assumptions. The LEI Team's role also included naming and reworking underlying narratives, for example, the tendency to attribute ‘positive outcomes’ to treatment adherence and to minimize accounts of harm. These forms of work became especially visible in the revision of the foundational ‘Introduction to Psychosis’ training, discussed in a later section.

In ORC terms, although organizational change efficacy (i.e., the belief that the system had the supports and processes to carry out the change) was still developing, the team possessed strong internal change efficacy. Members shared a clear purpose and brought diverse professional experiences, including peer support practice, clinical training, research and management experience, and legal and advocacy backgrounds. Recruitment emphasized lived experience as well as interpersonal capacity and role‐relevant skills, including the ability to engage controversial topics and collaborate despite differences. This was essential because no single perspective could represent the whole service population; we deliberately sought people with different relationships to psychosis and treatment (such as institutionalization, direct service in CSC clinics, peer support roles, community/survivor work). Not every perspective was represented on the initial team, however, missing perspectives informed recruitment priorities and were brought into the work through advisory council participation.

The work of the LEI Team was personally demanding. Members brought histories of psychosis, hospitalization, coercive or ambivalent treatment experiences, and ongoing encounters with stigma. Integrating these perspectives into a statewide technical assistance program meant that routine meetings about deliverables could quickly become spaces where unresolved injustices and structural harms surfaced; these are topics that could not be rushed without risking further harm.

This intensity was amplified by the institutional context. Large medical and behavioural health systems carry particular weight for many people with lived experience of psychosis. Compared with roles situated in community‐based or peer‐run settings, participating at the statewide TTA level required stepping directly into systems that can feel like the ‘belly of the beast’—institutions positioned as authorities over care rather than collaborators in it. Whether in leadership or advisory roles, returning to this space involved navigating cultures that have not recognized experiential knowledge as legitimate. As a result, responsibility for sensemaking and relationship management typically fell to individual LEI Team members in their own time.

## 7 Early Implementation Activities: Persistence and Cooperative Behaviour

Although the Lived Experience Integration Team's mission and intended role were presented in program forums and the team was frequently invoked, there was no program‐wide mechanism for determining when or where the team should be involved; its role was often negotiated as needs arose. As a result, LEI involvement unfolded inconsistently throughout the TTA. The team was invited into some projects during early conceptual planning and into others only after major decisions and materials were already in place. This variability limited the team's ability to shape EPI‐CAL's activities and revealed a system where structural readiness for lived experience integration was still emerging.

In response, the team focused on what it could control: building internal stability and predictable ways of working. Because the LEI Team was asked to bring perspectives informed by lived experience into multiple TTA program activities, one early priority was to avoid positioning any one member as representative of the range of experiences the team sought to include. Establishing internal review structures became a practical strategy for functioning amid shifting timelines and variable inclusion points. Requests for consultation or document review were routed through a standard intake form and assigned to pairs of members for closer review. Those pairs brought their impressions back to the team, where we attempted to reach consensus before the team lead relayed written feedback. This process allowed multiple perspectives to be considered before feedback was shared externally and reduced reliance on any one person as the team's representative voice. We worked toward checking our own blind spots so that divergent and critical perspectives could surface before feedback left the team. From an ORC perspective, these practices strengthened the LEI Team's own change efficacy in advance of fully aligned organizational processes.

The volume and diversity of requests demanded substantial coordination. The LEI Team met up to four times weekly to manage different strands of work: focused review/consultation meetings, sessions on peer support practices in Coordinated Specialty Care, advisory council preparation, and meetings devoted to research initiatives and mentorship. Subgroups split off to join project‐specific meetings with other training teams and clinics. In ORC terms, this suggested high collective capacity within the team, operating inside an environment where inclusion points and expectations were still being negotiated.

Over time, the team organized itself into a core leadership group—staff and a small number of contractors typically working at least 10 h weekly (Table 1)—supported by a peripheral group of contracted consultants who contributed on a more flexible basis. This arrangement accommodated different levels of commitment and availability and maintained a defined route for input into statewide activities. Additional contractors functioned as an independent advisory group that set its own priorities based on member input and operated alongside (rather than within) the LEI Team's operational structure.

## 8 Implementation Activities and Achievements

Despite EPI‐CAL's early phase of organizational readiness, the LEI Team's role expanded rapidly as recognition grew of the need for experiential perspectives across multiple components of Coordinated Specialty Care. Over the first 2 years, we fielded 68 consultation requests from every TTA training and administrative team (Figure 4). These requests ranged from brief language checks to multi‐stage collaborations on training development, workflow design, and questions about how to structure roles for people with lived experience. Even amid evolving structural supports, experiential knowledge became a routine part of shaping work in the TTA ecosystem.

**Figure 4:**
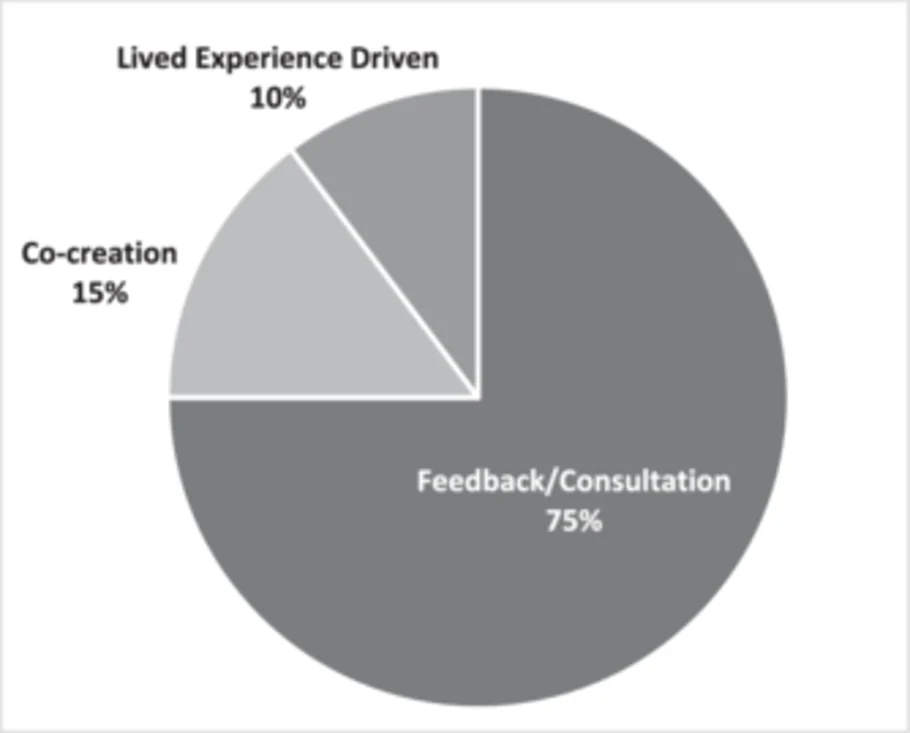
Completed Lived Experience Integration (LEI) Team requests during the first 2 years of implementation (*N* = 68), by level of involvement. Levels indicate LEI Team‐driven work, co‐creation (shared development) of materials with other EPI‐CAL teams, or feedback/consultation on materials developed by others.

Naturally, a substantial proportion of requests involved refining training content and strengthening the representational accuracy of examples and narratives, aligning with TTA workforce needs related to culturally responsive care and trauma‐informed practice [30]. Teams sought guidance on whether examples in trainings were consistent with service user experiences and whether the language used was likely to land as intended. This regularly extended beyond surface‐level language edits into deeper work of adjusting tone and framing. One significant effort centred on revising the foundational ‘Introduction to Psychosis’ training, one of the first modules that clinicians encounter when joining the EPI‐CAL network. The LEI Team collaborated with training developers to review how psychosis was portrayed and to flag descriptions that risked reinforcing stigma. Through iterative discussion, the team contributed revisions that better conveyed the range of psychosis‐spectrum experiences and the conditions under which distress emerges; this altered the tone of the module in ways that learners described as respectful and clinically useful. The process also helped the training team grasp the transformational impact of working alongside people with relevant firsthand experience and gave them an opportunity to work through the deeper issues the LEI Team had been highlighting. For LEI members, this project was especially meaningful as it allowed the team to frame the initial narrative through which clinicians encounter psychosis.

Many requests focused on everyday clinical tools and processes, including referral and assessment workflows, peer documentation templates, resource screening tools, strategies for running support groups, and the language used in data collection systems. Even small changes carried significant implications. For example, review of an Adverse Childhood Experiences assessment item on parental mental health identified wording that could unintentionally imply blame. The team recommended revisions to reduce stigma and better align assessment tools with program values.

A third category of requests involved scope of practice for nonclinical roles, including peer supporters, and workforce design within clinic teams. Programs sought guidance on how peers should be trained and integrated into multi‐disciplinary processes, and whether specific tasks like diagnostic screenings were appropriate for nonclinical staff. These consultations required balancing fidelity to established clinical standards with workforce constraints and high referral volume. LEI Team perspectives contributed to clearer expectations and more intentional integration of peer workers in myriad roles, including recognition that peers often function as bridges between service users and clinicians in ways that are not always formally defined.

Several requests centred on questions of connection and meaning‐making for youth with early psychosis, including how to identify supportive communities and interpret emerging language used by youth in care settings. These consultations informed guidance on communication practices and public‐facing materials, helping the program refine how it communicates with and about service users.

Finally, the LEI Team launched initiatives of its own. These included creating an advisory council to establish regular routes for service user input, facilitating an online support and resource‐sharing community, and initiating peer‐led research. The team presented an introductory peer support training and co‐developed additional trainings with other TTA teams, and facilitated multiple sessions led by an internationally‐recognized disability rights advocate. These initiatives expanded opportunities for people with lived experience to shape work within EPI‐CAL and demonstrated how their leadership can guide implementation and system culture when meaningful avenues for contribution are available.

## 9 Tensions and Lessons Learned

Although the LEI initiative generated substantial activity and influence, tensions pointed to change readiness gaps (Figure 5). The team commonly encountered misalignment between stated values and operational structures and roles, as well as variable receptivity to perspectives from people with lived experience.

**Figure 5:**
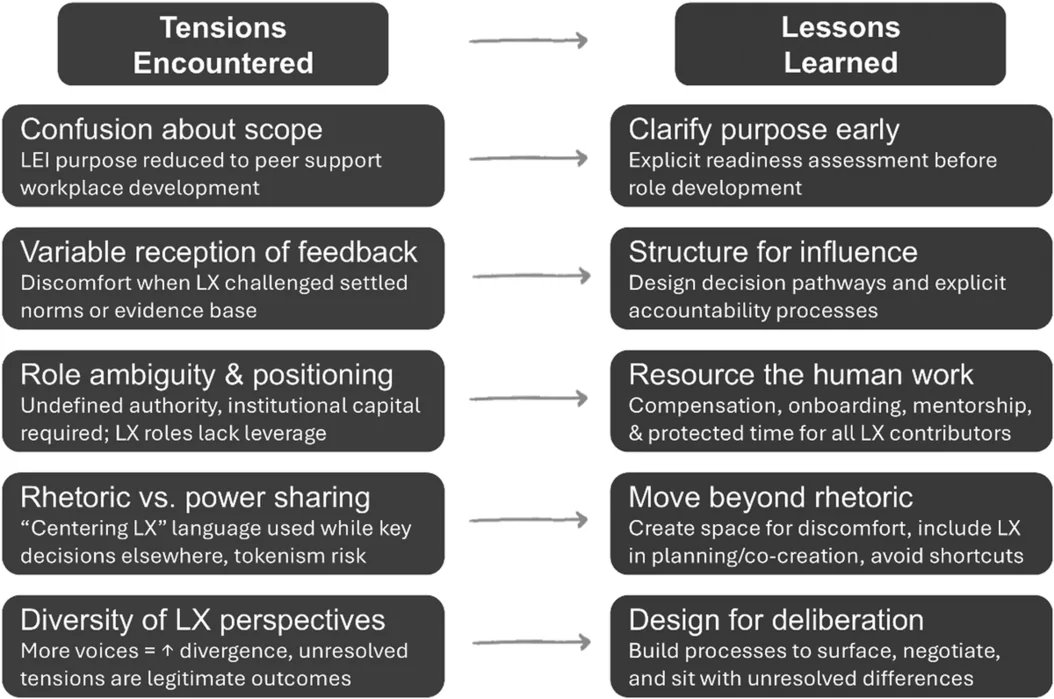
Summary of tensions and lessons learned during early Lived Experience Integration Team implementation.

**Early Confusion About Team Scope and Purpose.** A recurring challenge involved confusion about the purpose and scope of the LEI initiative. Across the TTA program, some colleagues assumed the effort centred on peer support workforce development. Peer support is one meaningful avenue for involvement of people with lived experience, but the scope developed by the LEI Team was broader: to bring critical perspectives into training development, advisory structures, research conceptualization, and program‐wide planning. This required addressing specific practices as well as deeper assumptions and subtext shaping how service users and recovery are conceptualized in early psychosis care.

Early misunderstandings of this scope created practical strain. The TTA did not have a distinct peer support training team or materials before the Lived Experience Integration Team was established. As Figure 2 illustrates, major CSC training functions were represented as separate teams, whereas LEI was positioned centrally to work across them. Nevertheless, LEI assumed responsibility for developing peer support training, and though it was viewed as an appropriate role, the concern was that this additional function could become conflated with the team's greater responsibility for lived experience integration throughout program operations. These dynamics pointed to an incomplete organizational understanding of the different forms of work required to incorporate lived expertise. As we often emphasized, the TTA could not credibly encourage clinics to centre these perspectives without attending to how they were incorporated in its own structures and practices.

**Variable Reception of Feedback.** Feedback from the LEI Team was generally received positively, but reception varied depending on the nature of the concerns raised. Although the team offered input through routine professional critique, comparable to ad hoc academic or clinical review, some colleagues showed surprise or discomfort when concerns were raised about language, clinical assumptions, curriculum framing, or the implicit narratives embedded in training materials. At times, reactions were communicated indirectly or outside of group discussion spaces, limiting opportunities for collective processing. These moments tended to arise when feedback concerned established, evidence‐based practices already in use, rather than areas still under active revision.

In ORC terms, these reactions suggest uneven change valence across teams, with stronger endorsement for symbolic or consultative forms of involvement by people with lived experience than for feedback that challenged established assumptions or redistributed narrative authority. These reactions do not necessarily indicate resistance; instead, this pattern illustrates a predictable change readiness gap. Without structured and supported processes for receiving this input, feedback that questions foundational norms is more likely to be personalized or deflected. This dynamic aligned with one of the LEI Team's early assertions: ‘If we are doing this work right, all of us will be challenged’.

**Role Ambiguity and Structural Positioning.** Several challenges arose from uncertainty about how the Lived Experience Integration Team fit within the TTA structure. Team members retained the supervisory relationships associated with their primary positions and organizations, while the LEI lead coordinated the team's work without formal supervisory authority over its members. The team's work crossed training and administrative streams without clearly defined authority or decision pathways, complicating decision‐making. This left expectations for the team's role dependent in part on the particular project and relationships involved.

An additional insight concerned the organizational position of the individual asked to launch and lead the effort. In our case, the system more easily legitimized a lived experience‐identified leader who had already accrued institutional capital. Holding a research faculty role provided sufficient standing to negotiate expectations between teams, advocate for other contributors with lived experience, and participate in meetings where program‐wide decisions were made. However, much of the labour required to develop and lead the LEI initiative fell outside conventional academic productivity metrics [27]. Individuals with lived experience who occupy such positions are rare, and when lived experience‐identified leaders are positioned in more junior or contingent roles, they have less organizational leeway to set boundaries or influence planning. People closest to the population being served, particularly young people in early stages of psychosis, are the least likely to be formally recognized by academic or clinical systems. Structural reliance on institutionally‐endorsed individuals to represent these perspectives therefore narrows whose voice is heard. Without intentional design, organizations risk reproducing this skew while appearing inclusive.

**Power Dynamics and the Limits of Rhetoric.** A related lesson involved the gap between language about ‘centreing’ lived experience and the actual distribution of power. Within the larger program, phrases such as ‘service user‐led’ or ‘peer‐centred’ were sometimes used aspirationally, even when people with lived experience were limited to consultative roles. This created risks of tokenism, as a small number of lived experience‐identified staff could be presented as evidence of meaningful change while key strategy decisions remained elsewhere.

There were also temptations to seek forms of involvement that were ‘easier’ for the organization to accommodate: preferentially engaging those already aligned with existing models of care, or amplifying stories that confirmed idealized narratives about recovery and service success. At times, this translated into expectations that the LEI Team function primarily as ‘language police’ despite efforts to clarify that this was not the team's core purpose. In practice, we learned that shortcuts to inclusion undermine both trust and impact. A meaningful LEI structure depended on making room for discomfort and stories that challenge dominant service narratives, instead of consolidating lived experience into a single, agreeable voice.

**Diversity of Perspectives and the Need for Deliberation.** A further lesson was that bringing more people with lived experience into a project reliably increased the diversity of viewpoints, not their convergence. Even within the LEI Team, members differed in their views on diagnosis, medication, coercion, and the value of early intervention for psychosis. Members also differed in how they made meaning of and described their own experiences, and these understandings could change over time and inform how they approached the team's work. When perspectives did not align, there was no obvious rule about whose experience should carry more weight, so we facilitated discussion to identify areas of agreement and conveyed remaining points of tension plainly in our feedback to collaborating teams. Disagreements were productive but time‐consuming, and they challenged assumptions that lived experience could be treated as a single, stable category. For EPI‐CAL, this meant that building readiness for lived experience integration could not be reduced to simply adding a fixed number of peer roles. It required creating processes for surfacing, negotiating, and at times sitting with unresolved differences among people who all had legitimate claims to being directly affected by early psychosis care. Further, the organization needed capacity to bring these differences into the larger conversation, whether that be in the development of trainings or policies for implementation of EPI‐CAL practices. It challenged the organization to hold and make transparent the conflicts in the field that needed discussion at all levels.

**Integrating Lessons Across Tensions.** These tensions reveal a recurring pattern in which lived experience involvement was encouraged in principle but constrained in practice. Roles were invited without clear authority, and feedback was welcomed only insofar as it did not challenge settled norms or require shared decision‐making. In this context, ambiguity functioned less as a transitional phase than as a structural condition that increased workload and limited influence, particularly when diverse perspectives exceeded the capacity of existing processes to engage them meaningfully.

## 10 Building Future Readiness for Change

**Clarifying Purpose, Values, and Readiness.** Early implementation identified practical strategies for strengthening readiness for lived experience integration in similar settings. In ORC, informational assessment (i.e., the degree to which organizations understand what a proposed change will require) is a core determinant of readiness. An explicit readiness assessment prior to role and team development can help anticipate structural and resource needs, and should include clarifying the organization's intentions and values around this involvement, ensuring that operational plans align with *what* teams of people with lived experience are asked to do and *why* they are being included. Because these roles often involve complex personal and relational work, alignment between organizational values and the principles guiding involvement of people with lived experience is critical; without this foundation, role expectations may feel psychologically unsafe or misaligned even when structural supports are in place. Values‐centred work also creates space to recognize lived experience as a distinct form of knowledge, without positioning it as subordinate to clinical or academic expertise. Shared values provide a foundation for co‐developing structures that function effectively only when their underlying purpose is collectively understood. A non‐exhaustive list of guiding questions to support readiness assessment is provided in the online supplement.

**Creating Structures that Support Influence.** High internal efficacy within lived experience‐identified teams is a strength that can be amplified through structural support. Programs considering similar initiatives may benefit from designing roles and decision pathways in advance; early involvement of lived experience‐identified leaders can reduce misalignment and support consistent influence as initiatives develop [23]. Because redistributing narrative authority in established systems introduces discomfort and tension, organizations must be explicit about how input from people with lived experience is expected to impact decisions; receptivity may not emerge organically. Since groups organized around lived experience often work through ‘bottom‐up’, relational processes, institutional structures may need to accommodate team configurations and workflows that differ from customary clinical or administrative models while still meeting funding and contract requirements. This could be, for example, a collective style of ‘leadership’ as opposed to one point person or team lead. Clarifying these issues early can prevent overextension of a small group of lived experience‐identified contributors and make it easier for new members to understand how their efforts relate to organizational decisions and processes.

**Resourcing the Human Work of Lived Experience Integration.** To support sustainable lived experience involvement, organizations must explicitly resource the unique components of this work rather than treating them as incidental. Distinct functions should remain visible even when they are carried by the same people. The peer support work illustrates this problem, as the LEI Team carried a discrete training function that was not separately represented in the organizational structure. Beyond making workload visible, lived experience roles need intentional support for relational labour and the emotional processing that can accompany this work. They also need time for sensemaking when personal experience is brought into organizational decision‐making. Structured mentorship and onboarding that helps contributors understand the larger program and how their role connects to organizational decisions can help establish these roles as core organizational labour and distinguish them from informal contribution [23]. Appropriate compensation and authority must match the assigned responsibilities, and support cannot stop with the contributors themselves; in fact, resourcing must also extend to the teams receiving lived experience input. Without structured ways to receive and process feedback, particularly when it challenges established practices, responsibility for navigating discomfort defaults to lived experience‐identified contributors themselves. Providing dedicated time and resources for this work helps distribute responsibility more evenly and supports more effective uptake of lived experience perspectives. Finally, sustainability depends on resourcing over many years. Sustained funding for lived experience positions reduces precarity and strengthens continuity. Protected time for knowledge co‐production and iterative adjustment helps teams maintain cohesion and values‐centred work within programmatic constraints.

**Implications for Research.** Empirical studies examining how organizational positioning and resources influence the sustainability and impact of initiatives led by people with lived experience would strengthen the evidence base for implementation. Additional research could explore the invisible labour embedded in roles built around experiential knowledge and identify institutional supports that reduce strain and foster retention, for example by evaluating how different configurations of mentorship/supervision and role design affect burnout and perceived impact. Comparative studies across early psychosis care networks may clarify which models for involving people with lived experience (such as advisory, collaborative, or leadership‐focused) are most feasible and impactful in varying organizational contexts, particularly given the broad and still‐evolving ways involvement has been conceptualized [31]. Finally, validating tools to assess organizational readiness *specifically for lived experience integration* could help programs anticipate needs and design structures with resourcing that better sustains emerging leaders [32].

## 11 Conclusion

The early years of the Lived Experience Integration Team reveal both the promise and the complexity of building meaningful representation of service users within a large, fast‐paced TTA network. Founded to elevate the voices of people with lived experience of psychosis and to foster substantive involvement in all areas of early psychosis work, the LEI Team grew from one founder to five core members and several consultants with personal experience of psychosis‐spectrum conditions, shaping statewide CSC implementation by shifting narratives and strengthening accountability to the people served by early psychosis programs.

Viewed through the lens of Organizational Readiness for Change, early implementation of the LEI Team illustrated strong change valence and active initiation. In contrast, informational assessment of roles built around experiential knowledge and their organizational requirements was limited. Structural and resource readiness also developed unevenly over time. As a result, and as can be expected to occur when novel roles are introduced inside established systems, these roles were implemented before there was a collective understanding of how they should function.

As the initial 2‐year period drew to a close, the Lived Experience Integration Team entered a new era. The scope of the lead role had expanded beyond what could reasonably be held with a research faculty appointment, and the team decided to transition from a designated lead role to a collaborative project management model. Funding streams then changed and the organization began reassessing structure and supports for long‐term involvement of people with lived experience; that process remains ongoing. This moment of transition surfaced a harder truth. ‘Nothing about us without us’ is easy to affirm in principle, though translating it into day‐to‐day organizational practice is labour‐intensive and uncertain. At present, it appears that there are no clear, mature examples of lived experience integration being sustained inside large‐scale mental health systems. Whether EPI‐CAL represents an early step toward that possibility, or simply another instructive attempt, remains an open question.

The LEI Team illustrates how values‐driven leadership, strategic positioning, and shared learning can create openings for many voices within systems that have historically kept lived experience at the margins. We offer these reflections in the spirit of transparency and collaboration, and we invite further dialogue about how organizations can build structures that meaningfully support lived experience leadership in academic medicine.

## Author Contributions

**Stephania L. Hayes:** conceptualization, investigation, funding acquisition, writing – original draft, writing – review and editing, visualization, methodology, validation, formal analysis, project administration, data curation, supervision, resources. **Katherine C. Sanford:** conceptualization, writing – review and editing, data curation, project administration. **Alishia Dauterive:** conceptualization, writing – review and editing. **Naomi R. Jones:** conceptualization, writing – review and editing, visualization, formal analysis, data curation. **Shannon Pagdon:** conceptualization, writing – review and editing, visualization, formal analysis. **Maureen F. McSweeney:** conceptualization, writing – review and editing, visualization. **Josephine Lumaquin:** conceptualization, writing–review and editing. **Mark Savill:** conceptualization, writing – review and editing, supervision. **Tara Niendam:** writing – review and editing, project administration, supervision, resources, data curation, funding acquisition.

## Conflicts of Interest

The authors declare no conflicts of interest.

## Supporting information


Supporting File

## Data Availability

Data sharing is not applicable to this article.
