# Supplementary material for: More Than One Voice: Strengthening Organizational Readiness for Lived Experience Integration in Early Psychosis Services
Source: Health Expect. 2026 Nov 1;29(6):e70944. doi: 10.1111/hex.70944 (PMC13724204; doi:10.1111/hex.70944)
Supplement: Supplementary file 1 [file HEX-29-e70944-s001.docx]

**Online Supplement**

**Practical Readiness Questions for Lived Experience Integration**

This supplement provides practical readiness questions to accompany the case analysis described in the main article. The main text uses Organizational Readiness for Change to examine how the Lived Experience Integration Team developed within EPI-CAL from 2022-2024 and how organizational conditions shaped early implementation. The questions below are intended for organizations considering or developing lived experience integration within early psychosis services, training and technical assistance networks, research programs, or related mental health service systems.

These questions are not intended as a prescriptive model or formal readiness measure. Rather, they offer a structured starting point for examining whether enthusiasm for lived experience involvement is matched by practiced organizational values, role clarity, decision pathways, resources, and support for the relational work involved. They may also help organizations identify where additional infrastructure is needed through the implementation process, and can inform future efforts to develop more formal readiness assessment tools for lived experience integration in complex service systems.

The questions are organized around domains that emerged from the case analysis: purpose and practiced values; organizational receptivity; role clarity and authority; representation and equity; workflow and decision-making; mentorship and development; compensation and credit; and sustainability and accountability. This focus is consistent with broader workplace inclusion literature, which emphasizes both participation in the work group and recognition of people’s distinct contributions, and with trauma-informed organizational practice, which depends on predictable roles, transparent decision-making, and conditions that support different voices. These domains reflect the organizational conditions needed to support lived experience integration as an ongoing change process.

**Purpose and Practiced Values**

1. What purpose is lived experience involvement expected to serve in this organization?
2. What parts of the work will lived experience-identified contributors be asked to shape?
3. Do organizational practices match stated commitments to lived experience involvement?
4. Where do current workflows, timelines, or leadership habits conflict with the values being claimed?
5. Is the organization prepared for lived experience-identified contributors to raise concerns that challenge established narratives or preferred service models?
6. How will the organization ensure that terms such as “led,” “centered,” “co-produced,” “collaborative,” or “advisory” accurately reflect the level of power being shared?

**Organizational Receptivity**

1. Who are the established champions who can advocate for lived experience integration when concerns arise?
2. Do those champions have enough authority to influence decisions?
3. Are program leaders prepared to revise plans or revisit assumptions in response to input from people with lived experience?
4. How knowledgeable are staff and leaders about stigma, trauma, power, accessibility, and meaningful inclusion?
5. How will teams be supported when feedback from people with lived experience creates discomfort or defensiveness?
6. What prior efforts related to lived experience involvement, advisory structures, peer roles, or inclusion work have shaped the organization’s current readiness?

**Role Clarity and Authority**

1. What roles will lived experience-identified contributors hold in different parts of the work?
2. Are lived experience-identified contributors acting as consultants, contributors, collaborators, advisors, staff, leaders, or some combination of these?
3. What authority comes with each role, including the ability to shape program decisions before they are finalized?
4. Which decisions *require* input from people with lived experience before moving forward?
5. Which meetings are lived experience-identified staff expected to attend, and which decision-making spaces should they have access to?
6. Who has final decision-making authority when recommendations from people with lived experience are not adopted?
7. Who is responsible for ensuring that input from people with lived experience is considered, responded to, and documented?
8. How will lived experience-identified contributors be informed about whether their input shaped a decision?
9. If lived experience-identified contributors are described as leading or co-leading work, what concrete power do they have?

**Representation and Equity**

1. How will the organization avoid positioning one person as the sole representative of lived experience?
2. How will multiple lived experience perspectives be included, especially when those perspectives conflict?
3. How will the organization seek input from people who are less visible in academic, clinical, advocacy, or professional spaces?
4. How will the organization make room for people whose experiences challenge preferred narratives about treatment, wellness/recovery, or service success?
5. How will participation be supported for people with different levels of education, professional experience, communication style, access needs, and availability?
6. How will the organization avoid treating lived experience-identified contributors as interchangeable?
7. Are credentialed or institutionally-fluent lived experience-identified contributors being heard more readily than people closer to the service population being discussed?

**Workflow and Decision-Making**

1. How will requests for involvement of people with lived experience be submitted, prioritized, reviewed, and returned?
2. What information and/or supports do lived experience-identified contributors need before they can provide meaningful input?
3. What timelines are realistic for meaningful input/review?
4. How will people with lived experience be included before major decisions are already settled?
5. How will the organization respond when feedback from people with lived experience conflicts with clinical, academic, administrative, or funder priorities?
6. What process will be used when lived experience-identified contributors disagree with one another?
7. How will unresolved disagreements be communicated to the larger organization?
8. How will recommendations be tracked so they are not lost across meetings or deliverable cycles?
9. What happens when an idea is valuable but the organization lacks capacity to implement it?

**Mentorship and Development**

1. What mentorship will be available to contributors with lived experience?
2. Who will provide mentorship, and is that work included in someone’s funded role?
3. Will mentorship include project orientation, skill-building, access to professional networks, opportunities to lead, and support navigating institutional dynamics?
4. Will lived experience-identified contributors be paid for time spent in training, onboarding, mentorship, and professional development?
5. What training will staff need in order to work respectfully and effectively with lived experience-identified contributors?
6. When does the level of responsibility expected of a lived experience-identified contributor require a staff role with benefits rather than a short-term consulting arrangement?

**Compensation and Credit**

1. How will lived experience-identified contributors be compensated fairly and predictably?
2. Will compensation reflect time, role expectations, specialized expertise, and preparation demands?
3. Are payment and reimbursement processes accessible for contributors who cannot wait months to be paid?
4. What contributions qualify for authorship, acknowledgment, consulting credit, leadership credit, or other forms of recognition?
5. How will expectations about authorship and credit be discussed before work begins?
6. How will the organization avoid overstating involvement of people with lived experience in publications, presentations, reports, or grant materials?

**Sustainability and Accountability**

1. What would successful lived experience integration look like in this organization, and who gets to define that success?
2. Are roles and protected time for lived experience-related work included in budgets, scopes of work, staffing plans, and timelines?
3. Are funders aware of the resources required for meaningful lived experience integration?
4. What plan will sustain involvement of people with lived experience beyond short-term project cycles?
5. What happens if funding changes, champions leave, or program priorities otherwise shift?
6. How will lived experience-identified contributors raise concerns about the integration process itself?
7. How will the organization monitor whether involvement of people with lived experience is becoming symbolic or overly dependent on a small number of people?
8. Who is responsible for addressing concerns when lived experience integration is not functioning as intended?
9. What structures will support the emotional and relational labor involved in roles related to lived experience integration?
10. How will the organization evaluate whether involvement of people with lived experience shaped decisions, materials, workflows, or organizational culture?
